# Argentinian Mental Health During the COVID-19 Pandemic: A Screening Study of the General Population During Two Periods of Quarantine

**DOI:** 10.32872/cpe.4519

**Published:** 2021-03-10

**Authors:** Martín Juan Etchevers, Cristian Javier Garay, Natalia Inés Putrino, Natalia Helmich, Gabriela Lunansky

**Affiliations:** a Faculty of Psychology, University of Buenos Aires, Buenos Aires, Argentina; b Faculty of Psychology, University of Amsterdam, Amsterdam, The Netherlands; Philipps-University of Marburg, Marburg, Germany

**Keywords:** COVID-19 pandemic, mental health, Argentina, quarantine

## Abstract

**Background:**

Due to the COVID-19 pandemic, Argentina has been under mandatory quarantine. We have aimed to investigate the state of mental health of the Argentine population and the behaviours adopted to cope with mental distress during quarantine.

**Method:**

An online survey was conducted using a probabilistic sampling technique and stratified according to the geographic regions of the country. The survey covered days 7-11 (n = 2,631) and days 50-55 (n = 2,068) after compulsory quarantine. The psychological impact was measured using the 27-item Symptom CheckList (SCL-27), which provides a Global Severity Index (GSI). An ad hoc questionnaire registered problematic, healthy and other behaviours. Two network models were estimated using a Mixed Graphical Model. Data from the two periods were compared and analysed.

**Outcomes:**

Higher GSI scores and greater risk of experiencing mental disorder were found in Period 2 as compared with Period 1. The lowest GSI scores were associated with physical activity in both periods, and meditation and yoga in Period 1. Drug users reported the highest GSI scores in both periods. The Network Comparison Test confirmed a significant change in symptomatology structure over the two quarantine periods.

**Conclusion:**

This study showed that psychological symptoms and the risk of experiencing mental disorder increased significantly from Period 1 to Period 2. Network analysis suggested that the quarantine might have brought about changes in the relationships between symptoms. Overall results revealed the relevance of mental health and the need to take mental health actions upon imposing quarantine during the current COVID-19 pandemic.

## Abstract (highlights)

- The length of the quarantine is associated with increased psychological symptoms.
- The youngest, the ones with a low income and females reported the most symptoms.
- Physical activity is less associated with psychological symptoms.
- Access to mental health assistance is crucial to minimize the psychological impact of quarantine.

*Pandemics* are *epidemics* on a large scale which affect people in multiple countries and which sometimes, as is the case of the current COVID-19 pandemic, can spread globally (World Health Organization [WHO], 2010). There is a long history of fighting epidemics and pandemics (Huremovic, 2019). It is pertinent to highlight that, in the absence of adequate biomedical treatments, behavioural methods such as good hygiene practices and social distancing have been frequently implemented to reduce morbidity and mortality (Taylor, 2019). *Quarantine* is the restriction of movement of people who have been exposed to an infectious disease to determine if they have been infected and thus, reduce the risk of spreading the disease. *Isolation*, on the other hand, is the separation of people who have been diagnosed with an infectious disease from those who have not (Centers for Disease Control and Prevention [CDC], 2017; Hurtado & Fríes, 2010). Recently, *quarantine* has been implemented against the coronavirus disease 2019 (COVID-19) outbreak.

On March 3, Argentina confirmed its first COVID-19 case. School classes were suspended on March 16 with a strong non-mandatory recommendation for social isolation and, as of March 20, the mandatory quarantine came into effect; exemption was secured for health professionals, security and defence personnel, journalists and media professionals, and the food industry (Decreto Necesidad y Urgencia [Emergency Decree, Argentina], 2020). At the beginning of the quarantine, 30 cases and 3 deaths by COVID-19 were confirmed in Argentina (Ministerio de Salud [Ministery of Health, Argentina], 2020). The quarantine was enforced through police controls; city and town limits and provincial borders were closed, resulting in a 54.78% reduction in public transport usage (reaching 86%) (Google, 2020).

The *psychological effects* of quarantine have been studied in different past occasions and countries. From previous epidemic and pandemic studies, it appears that *the longest quarantine studied was a 21-day quarantine instituted* in 2015 in Liberia, a country in West Africa, on account of an Ebola virus outbreak. Three studies showed that prolonged quarantine was associated with symptoms of post-traumatic stress, avoidance behaviours and anger, among the most prevalent (Brooks et al., 2020). Also, an association between higher levels of psychological symptoms and low income, job and financial insecurity, and healthcare workers was also established (Holmes et al., 2020). Studies of recent and dramatic experiences with COVID-19 show similar or more serious results. (de Girolamo et al., 2020; Wang et al., 2020; Williams, Armitage, Tampe, & Dienes, 2020).

Although mental health aspects of the COVID-19 crisis play an important role in managing the pandemic, there is a pre-existing lack of mental health research studies in Argentina. Given factors such as quarantine duration, culture, politics and economic situation are unique to this study. This study, which aims to determine the psychological impact of these factors on the Argentine population, was carried out 55 days after imposition of mandatory quarantine and 72 days after the first confirmed COVID-19 case. More specifically, it intends to establish the impact of the pandemic and quarantine on psychological symptomatology in the Argentine population, and its relationship with certain behaviours, defined as healthy, problematic and others. We also aim to establish whether quarantine duration is related to symptom severity. Apart from investigating changes in symptom severity, we are likewise interested in the changes in symptomatology structure as well as in the relationships between symptoms and reported healthy and problematic behaviours as the quarantine period is extended. Network models are used for studying unique relationships between individual symptoms and the reported behaviours (Borsboom, 2017; Borsboom & Cramer, 2013). Furthermore, symptom network models show the unique associations between behaviours and symptoms, elucidating the possible pathways via which healthy or problematic behaviours can (negatively or positively) influence specific symptom development (Isvoranu, Borsboom, van Os, & Guloksuz, 2016). To this means, we will attempt to identify changes in symptomatology structure and symptom-behaviour relationships between the early and later quarantine phases by constructing a network model of psychological symptoms and behavioural variables.

## Method

### Study Design and Participants

We adopted a survey design to assess the impact of COVID-19 and quarantine by using an anonymous online questionnaire. The sample was probabilistic and stratified according to geographic regions of Argentina and its population distribution (see Table 1 and Table 2). The online survey was conducted on days 7-11 (from March 27 to 31, 2020) and days 50-55 (May 8 to 12, 2020) of the compulsory quarantine.

**Table 1:** Sample Characteristics of the Period 1 (Days 7-11 of Quarantine) and 2 (Days 50-55 of Quarantine)

Participants’ characteristics	Period 1 (*n* = 2631)		Period 2 (*n* = 2068)	
	*n*	%	*n*	%
Age
18-20	113	4	119	6
21-29	472	18	321	15
30-39	750	28	439	21
40-49	469	18	661	32
50-59	450	17	280	14
> 60	377	14	248	12
Gender				
Women	1210	46	1056	51
Men	1421	54	1012	49
Educational level				
Primary	143	5	80	4
Secondary	1056	40	777	37
Vocational	708	28	594	29
Higher	724	27	617	30
Income				
Low	1201	45	843	41
Middle	1281	49	1072	52
High	149	5.5	153	7

**Table 2:** Samples’ Geographic Distribution of the Period 1 (Days 7-11 of Quarantine) and 2 (Days 50-55 of Quarantine)

Region	Period 1 (*n* = 2631)		Period 2 (*n* = 2068)	
	*n*	%	*n*	%
Buenos Aires Metropolitan Area	1159	44	1011	49
Buenos Aires province	409	16	257	12
Córdoba	322	12	257	11
Rosario	269	10	178	9
Mendoza	246	9	157	8
Tucumán	226	9	111	5
Neuquén	–		132	6

### Psychological Symptomatology

The psychological impact of COVID-19 was measured using the 27-item *Symptom CheckList* (SCL-27; Hardt & Gerbershagen, 2001). The SCL-27 has been adapted and well-validated to the Argentine population (Castro Solano & Góngora, 2018). Two indexes were calculated: 1) the *Global Severity Index* (GSI -27), which is the total item mean scores; and 2) the *Risk of Mental Disorder Index*, which included participants who answered over 50% of the items (14 or more out of the 27 items in this instrument) with the options "quite" or "much”; these participants being thus regarded as at risk of developing mental disorders.

### Problematic, Healthy and Other Behaviours

Through an *ad hoc* questionnaire, problematic behaviours (alcohol, illegal drug and tobacco abuse), healthy behaviours (sports and physical activity, sex life and religious practice) and other behaviours (use of over-the-counter and prescription drugs, yoga or meditation practice) were registered. Associations with these behaviours and their changes during mandatory quarantine were analysed with GSI-27 indicators and the "risk of mental disorder" index provided by SCL-27.

### Procedures

After completing the informed consent process, participants filled an online questionnaire sent through a social network. It contained a socio-demographic section, the SCL-27 (Castro Solano & Góngora, 2018), and an ad hoc questionnaire on healthy, problematic and other behaviours mentioned below.

### Statistical Analysis

In order to compare the GSI-27 between the two periods, we conducted a paired-samples *t*-test. In addition, we compared risk of mental disorder and suicidal thoughts in the two periods through the *Z*-test for population proportions. In order to compare the effects of sex, age, and income on GSI in each period, we performed a one-way between-subjects ANOVA.

For the purpose of comparing the effects of problematic behaviours (tobacco, drug, and alcohol use), healthy behaviours (sports and physical activity, sex life and religious practice), and other behaviours (medication use, yoga or meditation practice) on GSI in each period, we carried out an independent-samples *t*-test. In an attempt to examine the relation between yoga practice and the risk of mental disorder, we performed a chi-square test of independence. Data were analysed using the Statistical Package for the Social Sciences (SPSS), version 18.0.

The network model was estimated with a Mixed Graphical Model (MGM), using the “mgm” implementation in the “bootnet” package in R (Epskamp, Borsboom, & Fried, 2018; Haslbeck & Waldorp, 2020). This model combined the use of categorical and Gaussian variables which allowed us to combine behaviours and symptoms into one network model. The MGM is not yet available for ordinal data, so we used the “Gaussian” option for the 5-point Likert scale symptom data, as suggested by Haslbeck and Waldorp (2020). Relationships between variables were statistically estimated based on conditional dependencies of the data. In order to test if symptomatology structure significantly changed from Period 1 to Period 2, we conducted the Network Comparison Test (NCT; van Borkulo et al., 2021) by using the “NCT” software package in R (van Borkulo, Epskamp, & Millner, 2016). The NCT compared the symptom networks from the two periods based on their structure and overall connectivity (i.e., the strength of statistical associations between symptoms). This test cannot be performed on mixed data, which is why we conducted it on symptom networks only containing the SCL-27 symptom data (i.e., without behaviours).

## Results

2631 participants completed the online survey in Period 1 and 2068 participants completed it in Period 2.

### Psychological Symptomatology

Firstly, it was evaluated if the psychological symptoms differed between Period 1 and Period 2. In addition, the risk of experiencing a mental disorder and suicidal ideation in both periods was estimated. A significant difference was observed in GSI scores, *t*(2067) = -50.664, *p* < .001, between the two periods; Period 2 yielding the highest score. We also identified a significant difference between the two population proportions according to the Mental Health Risk Index, *z* = 3.48, *p* < .01. During Period 1, 4.86% of participants were at risk of mental health disorder, while during Period 2, 7.2% of participants were at risk.

An independent-sample *t*-test comparing GSI values of individuals with suicidal thoughts and individuals without suicidal thoughts showed a significant difference in Period 1, *t*(2629) = 18.16, *p* < .001, (individuals with suicidal thoughts [*M* = 1.9, *SD* = 0.82] and individuals without suicidal thoughts [*M* = 0.81, *SD* = 0.61]). Important differences were also detected in Period 2, *t*(2066) =18.03, *p* < .001, (individuals with suicidal thoughts [*M* = 2.96, *SD* = 0.71] and individuals without suicidal thoughts [*M* = 1.9, *SD* = 0.66]). A *Z*-test for population proportions was performed between the two periods for suicidal thoughts (*ad hoc* question). Significant differences were found; Period 2 yielding the highest score (*z* = 3.28, *p* < .01, Period 1 = 4.22%; Period 2 = 6.53%).

Regarding sleep disturbances, Period 1 showed that 73.7% of the sample had sleep related problems. In Period 2, 76.06% of the sample reported sleep disorders. Concerning sex life, 43.97% in Period 1 and 44.39% in Period 2 reported sexual dissatisfaction. No significant differences were observed. See Table 3.

**Table 3:** Screening Symptomatology Comparing Samples of the Period 1 (Days 7-11 of Quarantine) and 2 (Days 50-55 of Quarantine)

Measure, index and symptomatology	Period 1 (*n* = 2631)	Period 2 (*n* = 2068)	*p*
Mean GSI-27 (*SD*)	0.85 (0.66)	1.96 (0.71)	< .001^b^
SCL-27 mental disorder risk	128/2631 (4.86%)	149/2,068 (7.2%)	< .01^a^
Suicidal thoughts	111/2,631 (4.22%)	135/2,068 (6.53%)	< .01^a^
Sleep disturbance	1,572/2,631 (73.7%)	1,939/2,068 (76.02%)	ns
Sexual life dissatisfaction	1,157/2,631 (43.97%)	918/2,068 (44.39%)	ns

*Note.* GSI-27 = Global Severity Index of SCL-27; SCL-27 = Symptom Check List-27. SCL-27 mental disorder risk = participants who choose score 3 or 4 in at least 50% of the items; ns = Not significant.

^a^*Z*-test. ^b^*t*-test.

### Age, Sex and Income

We compared GSI values with socio-demographic characteristics (i.e., age, sex, and income). The lowest GSI values corresponded to the eldest participants in the sample, in both periods, *F*(5, 2625) = 31.322, *p* < .001, and *F*(5, 12.88) = 26.67, *p* < .001. The highest scores corresponded to women, also in both periods: Period 1, *t*(2618) = 10.77, *p* < .001, and Period 2, *t*(2055) = 8.91, *p* < .001.

Lowest income participants reported the highest GSI scores as compared to middle and high- income participants in both periods: Period 1, *F*(2, 2349) = 29.65, *p* < .001, and Period 2, *F*(2, 6.82) = 13.45, *p* < .001). See Table 4 for post hoc analysis and descriptive results.

**Table 4:** GSI Post Hoc Comparisons Using HSD Test on Age, Sex and Income, in Period 1 and 2

Participants’ characteristics	Period 1: GSI-27		Period 2: GSI-27	
	*M*	*SD*	*M*	*SD*
Age				
18-20	1.05	0.06	2.41	0.80
21-29	1.02	0.03	2.18	0.73
30-29	0.96	0.02	2.01	0.74
40-49	0.85	0.03	1.90	0.66
50-59	0.69	0.03	1.84	0.67
60 or more	0.58	0.03	1.68	0.71
Sex				
Men	0.77	0.58	1.80	0.66
Women	1.00	0.71	2.10	0.73
Income				
Low	0.98	0.71	2.06	0.69
Middle	0.75	0.59	1.90	0.69
High	0.74	0.59	1.87	0.66

*Note.* In Period 1, post hoc comparisons using the Tukey HSD test indicated that the mean score for the 18-20 and 21-29 years old subgroups had significantly more symptoms than the 40-49, 50-59, and 60 plus years old subgroups. Also, the 40-49 years old subgroups had a higher GSI than the 50-59 and 60 plus years old subgroups. In Period 2, the 18-20 years old subgroup had significantly more symptoms than 21-29, 30-39, 40-49, 50-59 and 60 plus years old subgroups. The 21-29 subgroup had significantly more symptoms than 30-39, 40-49, 50-59 and 60 plus years old subgroups. Also, the 30-39 years old subgroup had a higher GSI than the 50-59 and 60 plus years old subgroups. The 40-49 years old subgroup had more symptoms than the 60 plus subgroup.

In Period 1, post hoc comparisons using the Tukey HSD test indicated that the mean score for the low-income participants had a significant difference with the middle and high-income participants. In Period 2, the low-income participants reported the highest GSI score. Low-income participants had a significant difference with the middle and high-income participants.

### Problematic, Healthy and Other Behaviours

With respect to problematic, healthy and other behaviours, lower GSI scores were found in individuals who did physical activity both in Period 1, *t*(2629) = -6.63, *p* < .001, and in Period 2, *t*(2066) = -6.46, *p* < .001. In a similar manner, lower GSI scores were found in those who practiced meditation in Period 1, *t*(2629) = -3.19, *p* = .001). Again in Period 1, lower proportions of participants in the Risk of Mental Health Index were associated with the practice of yoga, χ^2^(1, *N* = 2630) = 9.94, *p* < .01. Regarding religious practice, we did not find considerable differences.

Drug users reported the highest GSI scores in Period 1, *t*(2601) = 4.93, *p* < .001, and Period 2, *t*(2033) = 3.54, *p* < .001. Tobacco users showed higher GSI scores during Period 1, *t*(2629) = -3.76, *p* < .001).

Alcohol was consumed by 37.51% of participants (*n* = 987) in Period 1 and 41.15% of participants (*n* = 851) in Period 2. 27.43% (*n* = 271/988) of participants in Period 1 and 33.73% (287/851) in Period 2 referred that their alcohol consumption had increased. Differences were not significant.

Over-the-counter and prescription drugs were used by 33.33% (*n* = 877) of participants in Period 1 and 33.12% (*n* = 686) in Period 2. Differences were not significant. More participants used prescription drugs for coping with distress (anxiety, “nerves”, relaxation, sleep) in Period 2 than in Period 1, but we did not find a marked difference.

Considering mental health care, in Period 2, 14.02% (*n* = 290) of participants were in psychological treatment and 37.55% (*n* = 668) of responders that were not receiving mental health care considered that they needed treatment but pointed to difficulties in accessing mental health care systems.

### Network Analysis

**Figure 1:**
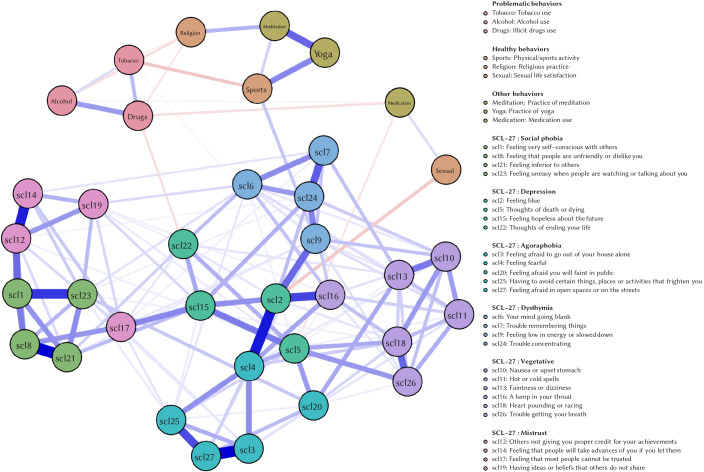
Estimated Network Model – Period 1 *Note.* The estimated network model includes the SCL-27 variables and behavioural variables for quarantine Period 1. The nodes in the figure represent the variables, and the lines between the nodes represent the edges, which encode the statistical associations between variables. The colour of the edges represents the nature of this association: blue edges represent positive associations; red edges represent negative associations. Thickness of edges represents the strength of associations.

**Figure 2:**
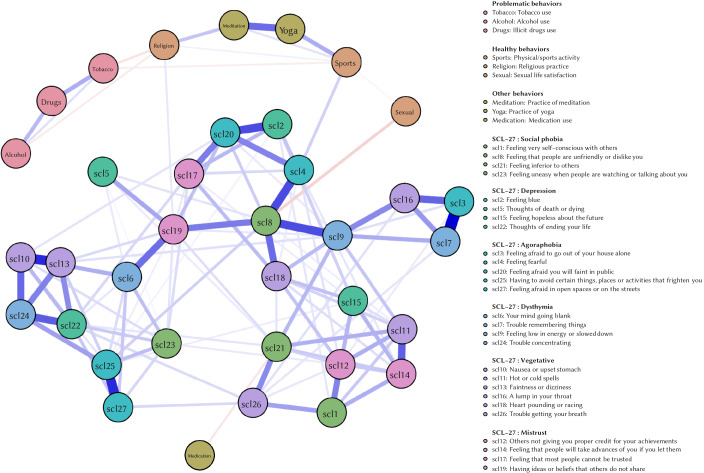
Estimated Network Model – Period 2 *Note.* The estimated network model includes the SCL-27 variables and behavioural variables for quarantine Period 2.

Figure 1 and Figure 2 show the estimated network models for both periods.

Regarding the structure of symptom network models, the network model for Period 1 shows that symptoms cluster together according to their domain: this means that the items designed to measure the same domain have indeed strong positive associations amongst each other.

However, this does not apply to the network model for Period 2. Here, symptoms no longer cluster together according to their domain and symptom relations are interchanged.

Results from the NCT confirm the change in symptom structure: the structure of the symptom networks changed substantially over the two quarantine periods (*p* < .01). However, the global connectivity of the symptom networks was not altered (*p* = .98). This means that associations between different symptoms changed significantly over the two periods, but overall associations between symptoms did not increase or decrease.

## Discussion

This study is limited in the sense that participants were recruited through a social network and completed an online survey; therefore, individuals lacking access to the Internet or an electronic device, or presenting more severe symptoms, have not been included in the sample (Pierce et al., 2020). This is particularly important in Argentina, as it is a country with high poverty rates (Instituto Nacional de Estadísticas y Censos [INDEC, 2019]). However, the number of registered cell phone users in Argentina exceeds its total population. Nevertheless, this study is a contribution to the understanding of the mental health impact of COVID-19 pandemic and its subsequent mandatory quarantine.

This study showed that symptom indicators notably increased as the quarantine was extended. In addition, there is an indication that the risk of mental health disorders is also increased. Whereas diffuse symptoms may require lower intensity interventions, deep seated psychological problems call for more complex interventions by mental health professionals. Individuals with mental disorders were identified as the most vulnerable group, and the literature endorses the need to approach this group with a more comprehensive evaluation (Duan & Zhu, 2020).

The percentage of participants having suicidal thoughts increased greatly from Period 1 to 2. This surge is correlated with the increase in clinical psychological symptoms and risk of mental disorder mentioned above. Although certain symptoms are expected to increase in such extraordinary circumstances, there is concomitant risk that increased mental disorders lead to pathological behaviours such as self-harm, suicide and domestic violence (Holmes et al., 2020). A recent US study on COVID-19 and suicide mortality reported the highest rates since 1941 (Reger, Stanley, & Joiner, 2020). Preventing suicide risk is a priority which requires immediate interventions and actions (Gunnell et al., 2020).

In regard to participant’s sex life, our findings were consistent with evidence in the scientific literature which reports higher levels of overall prevalence of psychological symptoms in women compared to men (Mazza et al., 2020). In addition to biology-based roles, women in Latin America exhibit greater levels of stress on account of the number of tasks they perform and the social pressure to which they are subjected, as well as their exposure to gender discrimination and violence (Economic Commission for Latin America and the Caribbean [ECLAC], 2020). In both periods, younger women reported more symptoms than older women. In Argentina, 35.5% of the general population and 42.5% of its youth live below the poverty line (INDEC, 2020). Young people are therefore more vulnerable, have greater job instability, and fewer resources in general. The pre-existing Argentine economic recession has been exacerbated by the adverse economic effects of the quarantine on the entire population. Indeed, our study confirmed that lower income sectors experienced higher risk of mental disorder. This population is more exposed to labour, housing and economic uncertainty, factors that can impede quarantine compliance. Hence, the official slogan "stay at home" was adapted to the reality of these vulnerable areas and became: "stay in your neighbourhood". For the middle class sector, monthly rent fees became an additional stressor in the face of financial uncertainty and, in fact, during the quarantine, the Argentine government issued a controversial decree for the suspension of payment of rental fees and yet another decree which prohibited dismissals. Higher income sectors presented less symptoms possibly resulting from its access to greater resources to face the mandatory restrictive measures for the quarantine period and the loss of income during the pandemic. Besides, this social sector has access to health insurance or prepaid health coverage, which can prove crucial during the COVID-19 crisis.

According to our findings, more than half of the population did not engage in the healthy behaviours considered. Furthermore, as quarantine duration kept getting moved, a tendency to dismiss them was observed. It should be borne in mind that the mandatory quarantine during the period studied only allowed people to go outside their homes to get food and medicines. In addition, given that sport facilities and recreational areas remained closed, the population was forced to seek more restrictive alternatives such as video tutorials, online learning and workout classes in small spaces at home. Despite the fact that healthy behaviours could decrease the emotional impact of quarantine (e.g., those who did physical activity showed less psychological symptomatology in both periods), only a small percentage of the population resorted to these protective conducts, and this became accentuated as the quarantine progressed. Furthermore, the decrease in healthy activities can also be explained as a consequence of the changes in psychological symptomatology. The network analysis conducted provided an insight into the specific relationships between symptoms and behaviours. Domain-specific symptoms clustered together during the first period, but were significantly interchanged during the second period. This means that quarantine might have changed the symptom relationships which govern the specific symptomatology from which participants might suffer. Although there was no significant increase in global connectivity (i.e., associations between the symptoms of the network as a whole did not increase), this change in symptomatology structure, where the symptoms decreased in their domain-specific clustering, might indicate a worsening in symptomatology. Decrease in model fit regarding underlying symptom clusters has been related to a worsening of depression symptoms (Elhai et al., 2013). However, future research should focus on the implications of change in symptomatology network structure on symptom severity.

Sleep disturbances affected about 75% of participants in both periods of this study. Sleep problems are highly prevalent in both anxiety disorders and depression. Decreased physical activity and low exposure to sunlight in large cities alter sleep cycles. Over-sleeping was the most frequent sleep disturbance recorded in Period 1 of the study, while insomnia predominated in Period 2.

Regarding sex life satisfaction, almost 45% of participants in the present research reported that their sex life worsened during both Period 1 and 2. In comparison to the previous year, 35% considered that their sex life had deteriorated (Etchevers, Garay, Castro Solano, & Fernández Liporace, 2019). Sexuality is regarded as a healthy behaviour, together with physical activity and social life. Diminished sex life is associated with discomfort rates and widespread social restriction. Mandatory quarantine hinders sexual encounters for single or divorced / separated persons. It is to be expected that once the quarantine is over, these bonding difficulties will persist out of fear of contagion. Even in consolidated couples, human sexuality can be explained in the tension between presence and absence, which increases fantasy and desire. However, this item should be regarded with caution, because the great majority of respondents preferred not to provide an answer.

Our results showed that alcohol consumption increased as the quarantine progressed. The same was not observed with respect to tobacco or illegal drugs. Consumption of substances constituted one of the problematic behaviours adopted to deal with psychological distress. Although they provide relief by altering the effects of neurotransmitters, thus producing feelings of pleasure or sedation, prolonged use eventually results in general health deterioration.

About 40% of participants reported the need for mental health treatment but pointed out to barriers to access mental health care. Among the reasons for this, they stressed personal financial problems together with a set of barriers associated with lack of medical coverage and lack of response from nearby health centres. Additionally, partial closure of mental health services, which provided only emergency consultations, together with the fact that clinical psychologists have not yet been authorized to resume face-to-face therapy sessions, made it even more difficult for the population to get access to psychological care. To the best of our knowledge, like it was discussed (Andersson, Berg, Riper, Huppert, & Titov, 2020), the problems that can be effectively addressed through distance modality (i.e., tele-psychiatry or tele-psychology) and there is evidence that digital psychological interventions are moderately effective in Low-Income and Middle-Income Countries according to a recent meta-analysis (Fu, Burger, Arjadi, & Bockting, 2020). Although the number of professionals adequately trained in this modality in Argentina have yet to be determined. The percentage of the population having the digital resources to access these approaches has not been established either. Improving the population's access to mental health care is a priority at this point in the quarantine. Our findings emphasize the need to improve monitoring of the psychological impact of the quarantine and pandemic, and to evaluate crisis interventions or approaches and face-to-face and non-face-to-face treatments in order to identify and implement optimal models. Likewise, it is essential to identify the degree of psychological support required by health care workers on the front line and its accessibility since this population is at greater risk of suffering psychological consequences.

The general results of this study show the relevance of mental health and the need to take action to protect it when implementing mandatory quarantine measures during the COVID-19 pandemic. Increased psychological symptomatology and the risk of mental disorder can in turn increase alcohol consumption or other risky behaviours for oneself or others, and medium-term quarantine compliance depends on the level of understanding and emotion regulation ability of the quarantined population. As the COVID 19 pandemic continues to sweep the world and mandatory quarantine in Argentina is extended, more methodologically rigorous studies need to be conducted in order to determine how to reduce their impact on mental health.
